# Women and Nerves

**Published:** 1890-10-25

**Authors:** 


### Turning Over New Leaves.

### Women and Nerves.

We are afraid that books like "The Modern Malady,"* which are not scientific and accurate enough for the student, can do nothing but foster curiosity in a medicine-loving but non-medical public. In this book the author, with an evidently very slender knowledge of physiology, endeavours to define and account for the various phases of nervous exhaustion, a task which it is allowed, over and over again in these pages, the medical profession has failed to accomplish. It is very easy for anyone to write or speak glibly about "nervous disease," "nerve deterioration," "nerve exhaustion," or "neurasthenia"; but what does the author mean by such terms, and what idea do they convey to the public generally? We may safely assume a somewhat more vague and shadowy notion than the medical man possesses who is nevertheless so frequently accused of knowing nothing about nerve trouble, and of labelling all obscure nervous diseases "hysteria." We have read the book with an unbiassed mind and an honest wish to learn what could best be done, in the first place to avoid neurasthenia, and in the second to get rid of it when acquired, but our attempt has been fruitless. Those reading this book without independent knowledge would carry away from it the conviction that every other woman one meets either suffers from neurasthenia, or, if she does not already, will do so presently. It is quite a relief to feel confident that this is not the case; that neurasthenia patients are the exception and not the rule, and that, happily, the large majority do still possess the *mens sana in corpore sano*, which is so essential to the welfare of the human race. We are not, however, the less sorry for the author, who seems to have come in contact with a quite unusually large number of nervous people.

There is no doubt that neurasthenia, whatever it may be, is an accompaniment of modern life, with all its bustle and hurry and severe competition. Nowadays women undergo physical and mental exertion for which, in many cases, they are quite unsuited and quite forget how heavily handicapped they are in their competition on would-be equal terms with men, who, moreover, have taken many centuries of educational monopoly to attain their present intellectual status. Is it strange, then, that woman's nervous system should give way under this strain? Hence, what women need is some knowledge of the laws of health which they may put into practice readily, and especially to be taught that they must exercise ordinary common sense, and that ill-health can be and ought to be avoided; or, if inevitable, must be reduced to a minimum, and is not to be meekly accepted. If those who undertake to instruct women on matters of health would enforce such views, we should hear less of "hysteria and neurasthenia." Not that we would deny the existence of a great deal of real nervous trouble concerning which medical knowledge is still very imperfect; but we must have patience whilst men like Professor Ferrier and Dr. Hughlings Jackson are pursuing their investigations, by which the public at large are already greatly benefiting, and will do so still more in the future. It is unfortunately inevitable that every disease has its hecatomb of victims before the true remedy can be discovered, so complex is the organism called "man." It was not until thousands had fallen victims to small-pox that Jenner made his great discovery; and so it is with neurasthenia. The social evolution has been so rapid; women have now adopted a life so different from that which they led in past ages, and they make so many and such unexpected calls on their nervous systems, that it is no small wonder they cannot all bear the extra strain. It is not, however, by inveighing against doctors and nurses, and the treatment pursued, that any good will be done in averting that ruin of health which is threatening all women, according to our author, but by inducing them to practice ordinary prudence and foresight as regards their health, and for such advice we look in vain in "The Modern Malady."

The two objects apparently aimed at by the author are:—

1st. To show up massage and allied treatments as ridiculous for the relief of "neurasthenia."

2nd. To prove how heartless, and even cruel, are those who apply the treatment, and how ill-judged is the behaviour of the attendants and friends of the nervously afflicted.

As regards the first, we can only regret that the author's experience has been so woefully small in this matter. If, however, she has always had to undergo "painful" massage, it has been at the hands of an incompetent masseuse, and it is a pity that, considering the large number of skilled persons who exercise this art, she has not taken the trouble to select a really first-rate operator. Massage, as a form of medical treatment, prescribed by a physician and carried out by a competent masseur or masseuse, has never, in the large experience of the present writer, been characterised as "painful." Massage, like other medical treatment, should not be undergone without medical authority, and, of course, if the medicine-loving British public will prescribe for itself, and ignorantly handle edged-tools, if it suffers the natural consequences of such rashness and ignorance, has it a right to forthwith blame others for its disasters, and unjustly accuse the medical profession of not understanding its "case"?

With regard to the second accusation of heartless and even cruel behaviour of those who apply massage or are the attendants or friends of "nervous" persons, we think if the author had had more personal experience of the care of such patients she would not be so ready to condemn others. All doctors and nurses who have had any experience of "nervous cases" are in accord as to the extreme difficulties they have to encounter to control a woman who has lost all self-control—to meet her utter selfishness and total want of consideration for others—to hit the happy medium between indulging her morbid craving for a sympathy which she requires to be constant and abundant beyond the measure which she has a right to demand, and which is more than an ordinary human being is capable of sustaining. Is it, therefore, wonderful that the friends or relations or attendants of such people should sometimes lose patience and be stern, or even hard? We can well imagine a hysterical patient would not hesitate to accuse any attendant, who showed firmness, of downright brutality. It is alarming to see the extent of the moral degradation which follows uncontrolled emotions—the perverted sense of right, the untruthfulness and the cunning to which such persons can descend. No right-minded or just person would say a constable was harsh or cruel in preventing a would-be murderer or suicide from carrying out his purpose, even if he had to use force in the discharge of his duty. The would-be criminal has lost self-control, at least for the time, and has some defect in his moral character (whether inherited or due to education and surroundings makes no difference in the fact, though it may modify our judgment of the crime); similarly, hysteria being largely due to perverted moral sensibility, we have no right to exonerate the latter from blame, whilst we condemn strongly the former.

It is impossible in our limited space to touch upon all the points casually raised in "The Modern Malady," though there is one subject of great importance, *e.g.*, the chronic state of overwork of nurses, which we freely admit and deeply regret. The private nurse is too often left at the mercy of servants, who think it tiresome to have to take

* "The Modern Malady; or, Sufferers from Nerves." By Cyril Bennett, author of "The Massage Case." (Published by Edward Arnold.)

her meals up, and who, without the oversight of the mistress, seem to think "anything will do for nurse." Moreover, people forget that a night nurse is actively employed for ten or twelve hours, and that, even if it be night, she requires as many meals as she would during a similar number of hours in the day, and that to supply one meal is by no means sufficient.

In conclusion, the author relates an instance of "an erroneous impression conveyed to my brain through the ear," and she adds, "I was overdone at the time, and took the warning; that is to say, I rested." And if we may venture to give the advice, we would counsel the author of "The Modern Malady" to continue to rest from studying such matters as "neurasthenia," and turn her undoubted skill in writing to other uses for a time. It is not safe to dwell too long on such a subject as "nerves," and not wishing "Cyril Bennett" to become a victim of the modern malady, and fancying we detect a morbid note in this interesting book, we look forward to next reviewing a novel from her pen.

